# Supplementary material for: In Vitro Investigation of the Interaction of Avian Metapneumovirus and Newcastle Disease Virus with Turkey Respiratory and Reproductive Tissue
Source: Viruses. 2023 Mar 31;15(4):907. doi: 10.3390/v15040907 (PMC10144051; doi:10.3390/v15040907)
Supplement: Supplementary file 1 [file viruses-15-00907-s001.zip › Supplement Figure S1.pdf]

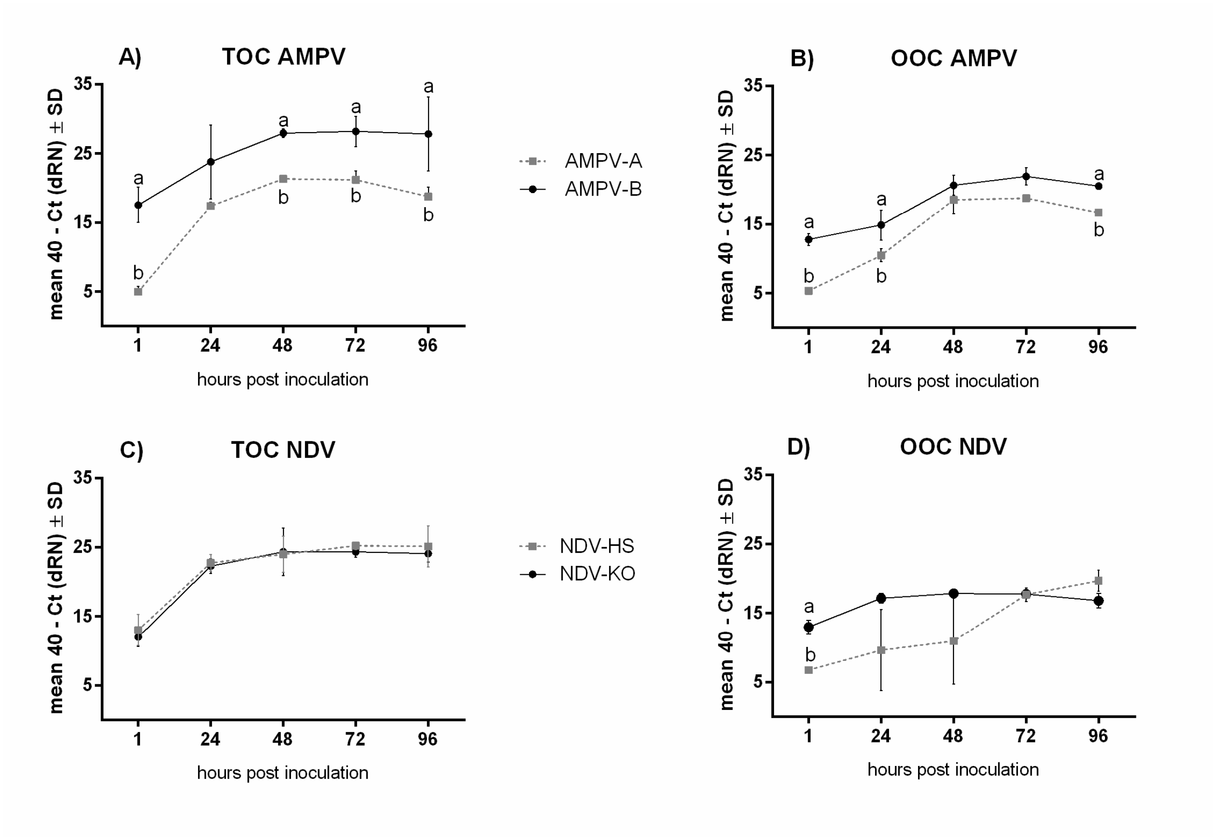

Supplement Figure S1. Comparative quantification of viral genome by qRT-PCR in TOCs (A,C) and OOCs (B,D) after inoculation with AMPV- (A,B) or NDV- (C,D) viral strains Per group, three to five rings were collected at 1, 24, 48, 72, and 96hpi and processed for viral quantification. Normalised data are presented as mean 40 - Ct. Error bars represent standard deviations (SD). Small letters indicate significant differences between compared viral strains at the same time point for each virus. Two-sample t-test. *P*-value < 0.05. Graphs represent data of one representative experiment.
