# Supplementary material for: In Vitro Investigation of the Interaction of Avian Metapneumovirus and Newcastle Disease Virus with Turkey Respiratory and Reproductive Tissue
Source: Viruses. 2023 Mar 31;15(4):907. doi: 10.3390/v15040907 (PMC10144051; doi:10.3390/v15040907)
Supplement: Supplementary file 1 [file viruses-15-00907-s001.zip › Supplement Figure S2.pdf]

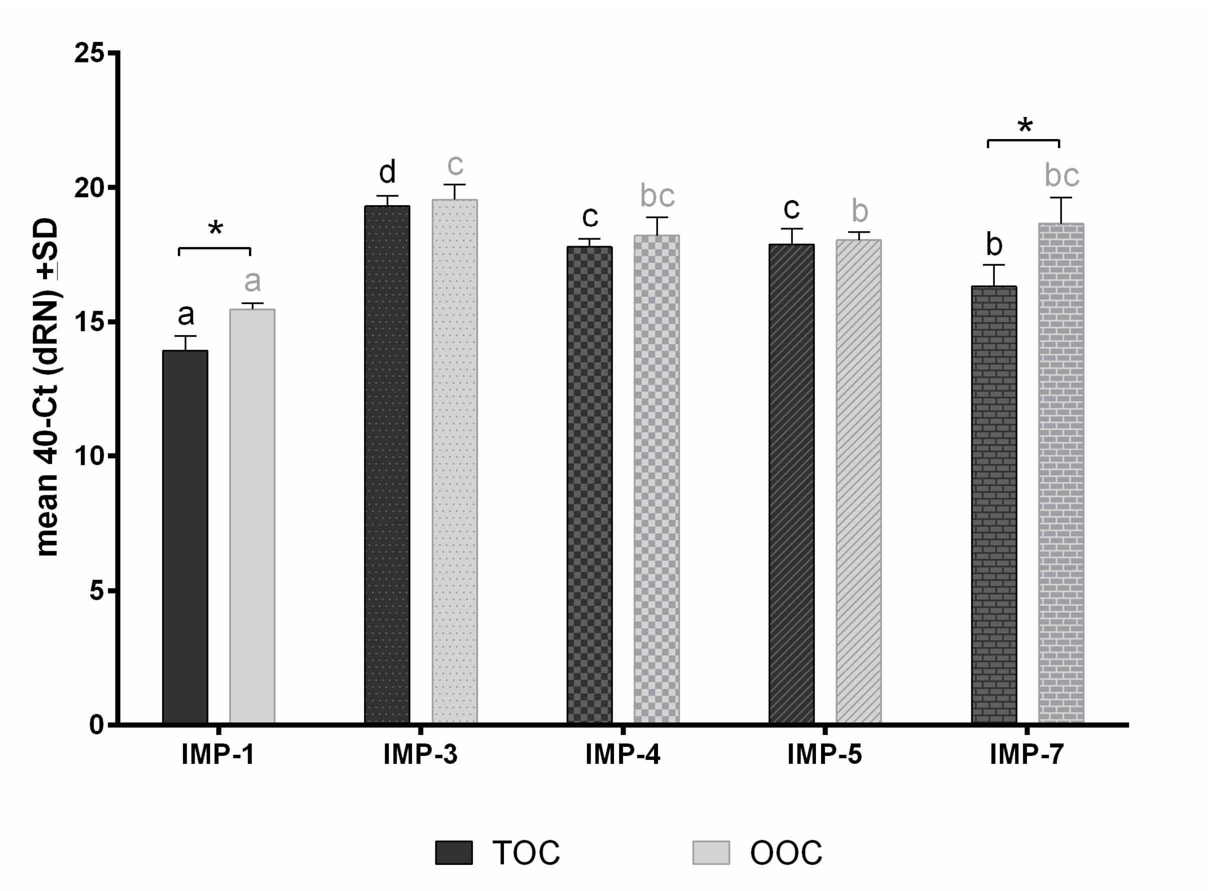

Supplement Figure S2. mRNA expression of different importin-α isoforms in TOCs and OOCs. The mRNA expression levels of importin-α 1,3,4,5 and 7 were detected in virus-free TOCs or OOCs at 24 hours after adding 100µl virus-free medium by qRT-PCR Data are presented as mean 40-Ct-values. Small letters indicate significant differences between the investigated importins either in TOCs or OOCs, Tukey HSD all-pairwise comparison test (ANOVA $p < 0.05$). Asterisks represent significant differences between both organs per importin, Two-sample *t*-test ($p < 0.05$). Graph represent data of one representative experiment.
